# Reducing Noise, Artifacts and Interference in Single-Channel EMG Signals: A Review

**DOI:** 10.3390/s23062927

**Published:** 2023-03-08

**Authors:** Marianne Boyer, Laurent Bouyer, Jean-Sébastien Roy, Alexandre Campeau-Lecours

**Affiliations:** 1 Department of Mechanical Engineering, Université Laval, Québec, QC G1V 0A6, Canada; 2 Centre for Interdisciplinary Research in Rehabilitation and Social Integration, CIUSSS de la Capitale-Nationale, Québec, QC G1M 2S8, Canada; 3 Department of Rehabilitation, Université Laval, Québec, QC G1 V0A, Canada

**Keywords:** electromyography, artifact, noise, interference, contaminant reduction, signal processing, denoising, filtering

## Abstract

Electromyography (EMG) is gaining importance in many research and clinical applications, including muscle fatigue detection, control of robotic mechanisms and prostheses, clinical diagnosis of neuromuscular diseases and quantification of force. However, EMG signals can be contaminated by various types of noise, interference and artifacts, leading to potential data misinterpretation. Even assuming best practices, the acquired signal may still contain contaminants. The aim of this paper is to review methods employed to reduce the contamination of single channel EMG signals. Specifically, we focus on methods which enable a full reconstruction of the EMG signal without loss of information. This includes subtraction methods used in the time domain, denoising methods performed after the signal decomposition and hybrid approaches that combine multiple methods. Finally, this paper provides a discussion on the suitability of the individual methods based on the type of contaminant(s) present in the signal and the specific requirements of the application.

## 1. Introduction

Electromyography (EMG) is a widely used method that records the electrical activity of muscles by measuring the electrical potential created when a muscle contracts (voluntarily or not). EMG signals can be detected directly using invasive electrodes placed in the muscle or as a superposition of signals gathered using electrodes applied at the surface of the skin. In the latter technique, known as the surface electromyography (sEMG), the signal is the combined electrical activity created by all motor units. When EMG was first developed, only intramuscular electrodes were used. However, inserting electrodes is an invasive procedure and implanted electrodes cause patient discomfort during muscle movement; sEMG was introduced to overcome these problems [1]. sEMG has several advantages over the use of invasive electrodes. First, the technique does not require a healthcare professional to insert the electrodes, which enables more widespread use in the research domain. In addition, the electrodes used are available at a low cost and some are even disposable. However, the sEMG also has disadvantages in that only the activity of surface muscles (i.e., muscles close to the skin) can be measured, and the method is more prone to noise because the electrodes are not inserted directly into the muscle. Thus, the signal sEMG measures acquires noise while traveling through the tissue [2].

EMG analysis is becoming increasingly important in many applications such as the clinical diagnosis of neuromuscular abnormalities, control of prostheses and other robotic mechanisms, muscle fatigue detection and quantification of force. In most of these applications, the EMG signal processing is performed under the assumption of an ideal signal. However, it is well known that myoelectric signals can be contaminated by various types of noise and artifacts, which can lead to a major misinterpretation of the data. Depending on their nature, contaminants can have several effects on both time domain and frequency domain features. The quality of the analysis in all applications is thus affected by the quantity and the characteristics of the contaminants. The fatigue analysis is traditionally performed using the mean and median frequency. Since these features are extracted from a frequency representation of the sEMG signal, any deformation of the spectrum due to an external cause could present a misleading picture of the fatigue state of the muscle [3]. In the same way, any EMG-based diagnostic may be inaccurate due to the presence of contaminants. This is the main reason prostheses that rely on myoelectric pattern recognition are not widely commercialized. Indeed, while the performance of these devices is good in controlled laboratory environments, their real-word performance is not entirely reliable due to the infinite sources of disturbance [4]. In prosthetic control, more than any other application, it is critical to maintain a robust interpretation of the intentions of the user, and for this, a clean EMG signal appears to be a prerequisite [5].

In the literature, many solutions have been proposed to attenuate the effects of noise and artifact contamination, such as skin abrasion to improve the electrode-skin contact surface, proper electrode and cable fixing, appropriate choice and use of electrodes (including bipolar vs. monopolar, choice of conductive gel to use with the electrode, electrode size, shape and inter-electrode distance) and appropriate electronics (e.g., amplification, analog filters, sampling rate, A/D conversion) [6]. However, in some applications, such as prosthetic control, the use of skin abrasion and other skin treatments to improve signal capture is not possible. Indeed, it would not be viable to ask the user to perform a skin abrasion every time they put on their prosthesis. Moreover, even assuming best practices, the collected signal may still be corrupted by noise and artifacts [7]. Accordingly, how can researchers asses this contamination problem and ensure they use a signal that has an adequate quality?

Another solution is to perform what is called a biosignal quality analysis after the acquisition. There are four types of signal quality analysis (1. Detection, 2. Identification, 3. Quantification and 4. Mitigation) [8]. Detection methods are used to detect contamination in a signal. Most of the time, a two-class classification scheme is used. The idea is to determine whether a contaminant is present in the biosignal. Identification distinguishes between contaminant types, while quantification provides an estimate of the severity of the contamination. Mitigation (henceforth referred to as contamination, reduction and/or denoising) attempts to remove or reduce contaminants in the recorded biosignal [9]. Each of the four analysis types is distinct, but they are often used together. Indeed, in most cases, we need to detect an artifact in order to quantify, identify and reduce it. In some cases, we also need to identify it to reduce it (by identifying the artefact, we can determine what contamination reduction method should be used). This allows us to achieve a better estimate of the power spectrum by eliminating only the contaminants that are present in the signal. Naturally, the goal is to reduce contamination while preserving the desired information in the EMG signal [10]. Therefore, it is important to ensure that no unnecessary contamination reduction method is performed on the signal. Moreover, in some cases, it is possible to use quantification features to identify/classify the artifact. In other cases, some quantification features imply that the user knows the type of artifact a priori (e.g., signal-to-motion artifact ratio (SMR), signal-to-powerline ratio (SPR), signal-to-ECG ratio (SER)). Conversely, quantification can also be used as a threshold to detect artifacts.

### 1.1. Review Objectives and Research Strategy

The aim of this paper is to review contamination reduction methods employed in the literature on single channel EMG signals. Specifically, we focus on methods which enable full reconstruction of the EMG signal without a loss of information. The term ’single channel’ refers to only one signal, meaning that, in this paper, only the methods that are meant to be applied to a single signal at the time are reviewed. This includes methods that are applied with mono-polar as well as bipolar electrode configurations. A number of methods tested using HD-EMG are also covered, provided that the denoising algorithm is applied to each channel separately. In addition, some of the methods proposed in the literature are performed on a processed version of the signal, such as its envelope. However, this review is also limited to contaminant reduction methods that are meant to be performed directly on the raw EMG signal. Furthermore, some of the methods found in the literature aim at denoising the signal for a single application. In these cases, researchers do not always ensure that the entire EMG signal is retained, as long as it helps in their own application. The focus of this paper is to review the contaminant reduction methods that allow for the total reconstruction of the EMG signal and can therefore be applied before any of the aforementioned applications.

Note that the aim of this review is not only to reflect the state-of-the-art regarding the contamination-reduction techniques but also to provide an overview of the main methods that have been proposed in the literature. This allows us to present the bigger picture of what has already been tested, what did and did not work well, and why.

Finally, only articles published in English or French were considered in the review process.

### 1.2. Content of the Article

This paper is organized as follows: Section 2 presents the types of contaminants that can be present in the EMG signal and the terminology used to describe them. Section 3 covers contaminant reduction methods, while Section 4 presents a discussion of their relative advantages and disadvantages. Finally, a conclusion is presented in Section 5 and a list of abbreviations appears in Section 6.

## 2. Contaminant Types

**Figure 1:**
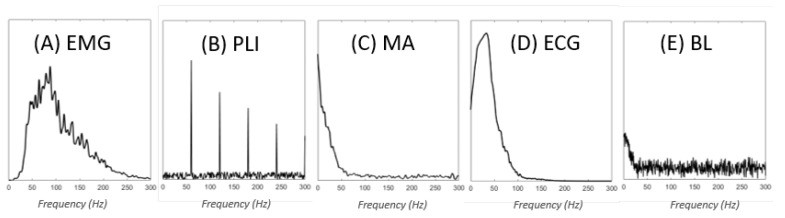
Power spectrum of the EMG signal (**A**) and of some of its contaminants: power line interference (**B**), motion artifact (**C**), electrocardiographic signal (**D**) and baseline noise (**E**).

There are several sources of EMG signal contamination. As presented in Figure 1, each contaminant has its own characteristics and affects the EMG signal in a different way. Among the main contaminants that generally cause signal processing problems, we can identify three categories: (1) baseline noise, (2) interference noise, and (3) artifacts. Other contaminants, such as amplifier saturation, ACD clipping and quantization can be avoided by using proper equipment. Therefore, they are not presented here.

**Figure 2:**
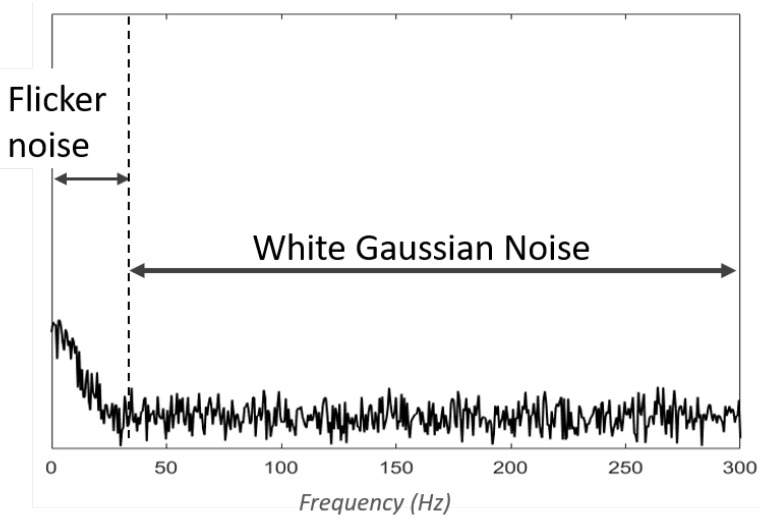
Baseline noise power spectrum.

Baseline noise (BL), also referred to as inherent noise, represents the signal detected by the equipment when the muscle is not contracted [10]. It includes noise caused by the amplification system, known as thermal noise, and is often modelled as white Gaussian noise (WGN). This means that it has a uniform power across the frequency band (Figure 2) and is normally distributed in the time domain, with an average time domain value of zero. The other component of the baseline noise is electrochemichal noise introduced by the interface between the skin and the electrode. This noise is caused by the impedance of the tissue at the skin-electrode interface, which acts as a low-pass filter. Consequently, in contrast to most noise sources, the electrochemical noise does not contaminate the signal in the normal sense. Instead, it has the effect of attenuating the signal of interest (>20 Hz), while keeping some undesired part of the signal (<20 Hz) as is. This results in a lower signal-to-noise ratio (SNR), which can also be modelled as the addition of flicker noise (1/f) or pink noise ($1/f^{n}$) (Figure 2) [11].

In contrast to baseline noise, which mainly arises from within the instrumentation system, the interference consists of unwanted signals (other than the EMG signal) captured by the equipment. A peculiarity of interference is that it manifests as a periodic signal. Interference can either come from other physiological signals, such as the electrocardiographic signal (ECG) and crosstalk from other muscles, or from ambient noise. ECG interference manifests in the EMG signal as a superimposed periodic signal whose energy may be greater than that of the EMG signal. Its frequency spectrum goes up to 100 Hz [12]. As for crosstalk, its spectrum is the same as the EMG, since it represents an EMG signal from another muscle. Ambient noise is caused by electromagnetic radiation that can arise from other equipment, such as electrical wires, televisions and radios [13]. Although other sources exist, the power-line interference (PLI) is the most problematic and widespread source of ambient noise; it is therefore also the most studied. PLI can be caused by the difference between the electrode impedances and the displacement current in the cables or in the body of the patient. Since it originates from power lines, PLI has a frequency centered at either 50 Hz (Europe) or 60 Hz (North-America) [14]. Very often, PLI includes harmonics of the fundamental frequency (i.e., multiples of 50 or 60 Hz).

Unlike other contaminants, artifacts do not necessarily present themselves as continuous waveforms (e.g., sudden peaks). Indeed, artifacts can be described as perturbations in the measurement of the EMG signal. The most common source of perturbation is body movement during recording, which can generate changes in the skin–electrode interface impedance and even give rise to fluctuations due to the motion of the cables [15]. These perturbations are called motion artifacts (MAs). MAs that come from the change in skin–electrode interface impedance are usually in the frequency range of 0–20 Hz, while MAs that arise from the motion of the cables can be up to 50 Hz [16]. Another possible source of artifacts is the electrical current induced by the electrical stimulation used in some applications to generate muscle contraction. Finally, other perturbations, such as poor electrode contact and electrode lift, can also create artifacts (mostly fluctuations of the DC component, i.e., <0.5 Hz).

### Other Terminology Considerations

Background noise (BN) refers to a mixture of various noises present in the electrode’s surroundings. It represents the noise present in a recording while the electrode is on the skin and the muscle is not contracted (i.e., no myoelectric signal is detected). In some references, this is referred to as ’baseline noise’ [17]. In this paper, however, the baseline noise represents only the inherent noise. A distinction also needs to be made between baseline wander (BW) and baseline noise (BL). BW is a low-frequency artefact (<1 Hz) caused mainly by respiration, body movements, poor electrode contact, and skin–electrode impedance. It is described as a long-term drift that can be observed through the variation of the DC components [18].

## 3. Contaminant Reduction Methods

Contamination reduction methods always represent a compromise between removing contaminants and preserving the EMG information [10]. The amplitude of a typical sEMG signal is between 0 and 10 mV across a frequency range of 10 to 500 Hz [19]. Since each contaminant has its own amplitude and frequency characteristics, the methods employed to reduce individual contaminants will vary accordingly.

### 3.1. Conventional Digital Filters

Taking into account the sEMG characteristics and the principal contaminants, researchers have used two principal filters for decades: band-pass filters (applied solely to the sEMG spectrum range) and band-stop filters (used to remove PLI). Band-pass filters can be separated in two parts: a low-pass filter and a high-pass filter.

**Low-pass filters:** As mentioned earlier, the sEMG spectrum comprises components of up to 500 Hz. Therefore, any signal with a frequency greater than 500 Hz is considered noise. Usually, this noise has the characteristics of white Gaussian noise. A low-pass filter with a cutoff frequency between 400 and 500 Hz is nearly always used to remove this noise. The cutoff frequency is not critical and can effectively be lower than the maximum frequency of the EMG signals because the portion of its energy over 350 Hz is very low [6].

**High-pass filters:** The high-pass filter’s cutoff frequency is more critical here because some EMG contaminants have overlapping spectra. For example, motion artifacts caused by movement of the body can be up to 20 Hz, while artifacts of up to 50 Hz can be caused by cables. To filter these contaminants most effectively without compromising the EMG signal itself, researchers have recommended several different cutoff frequencies (from 5–30 Hz) [20,21,22,23]. However, common ground has not been clearly established, likely because the optimal value depends on the application. More recently, ref. [10] compared three cutoff frequencies (10–20–30 Hz) used to filter motion artifacts and background noise from sEMG signals. They concluded that the cutoff frequency used should be based on both the application and the muscle studied. However, they also stated that cutoff frequencies lower than 20 Hz are not recommended, arguing that the proportion of the EMG signal is negligible compared to noise for frequencies below 20 Hz. On the other hand, ref. [24] highlighted that cutoff frequencies greater than 20 Hz may not be appropriate for fatigue analysis. Ref. [10] also recommended a minimal filter order of 2, which seems to be more generally accepted in the literature.

**Band-stop filters:** PLI is a principal contaminant of sEMG. Although some researchers argue that it can be assessed directly using the right sensors, it may still contribute to signal contamination. Further, in some cases, the use of active sensors is not always possible. The traditional means of removing PLI after acquisition is to use a narrow digital band-stop filter, such as a notch filter centered at 50 Hz or 60 Hz. As the name suggests, this kind of filter introduces a “notch” in the signal’s spectrum. Multiple notches can also be applied to remove its harmonics. An alternative to using multiple notch filters is the comb filter, which creates narrow rejection bands at every harmonic frequency [25]. However, the use of comb filters is not often reported in the literature. Band-stop filters (20–40 Hz) have also been reported in the literature to remove ECG by focusing on its maximum energy band [26].

Although these filters are quite powerful, their disadvantages have motivated the development of new techniques. Indeed, even if a band-pass filter can completely remove WGN outside its band-pass range, the portion of WGN that occurs within the EMG frequency band remains untouched. In the same way, spectral overlap also exists between the EMG signal and other contaminants, which obviously cannot be removed with high-pass filtering. For example, ref. [27] verified the effect of a 20 Hz high-pass filter in removing the ECG interference from an EMG signal and concluded that this was not a useful technique. Ref. [28] compared high-pass filters with cutoff frequencies of 10, 30 and 60 Hz in ECG removal; they advocated for the use of the 30 Hz cutoff. Further, digital filters can also introduce distortion in the remaining signal. Although this can be avoided by applying the filter, once forward and a second time backwards, this kind of treatment is not necessarily possible in real-time applications. Additionally, band-stop filters such as notch filters introduce a hole in the spectrum that removes not only the PLI component but also the EMG signal within this frequency band [6].

This has motivated the development of new contamination reduction methods to overcome these problems. The idea is thus to create an optimal filter, meaning a filter that tends to suppress noise while leaving the signal relatively unchanged. Consequently, two issues must be addressed. The first is the ability to minimize contamination, even within the frequency band of the EMG signal. The second issue is avoiding the signal distortion of the signal. Indeed, the relative contribution of all frequency components in the EMG signal should remain untouched [29]. Moreover, the denoising method should not introduce phase distortion, meaning there should be no observable time delay in the final signal. To design this kind of filter, we therefore consider that the measured signal is in fact a linear combination of two signals, the EMG signal and the noise (which can arise from any of the aforementioned contamination sources or a combination of them):

(1) x(t)=y(t)+n(t),

where x(t) is the measured signal, y(t) is the EMG signal and n(t) represents the noise. We can thus rewrite the equation as follows:

(2) y(t)=x(t)−n(t),

where the idea is to subtract the noise signal from the measured signal to obtain a better representation of the EMG signal.

### 3.2. Gating and Clipping Methods

The first and simplest method presented here is gating. The goal of this approach is to detect the artifact in the raw signal and to discard the time series containing these artifacts. Therefore, this method is only useful when applied to artifacts or interference that is not continuous over time. Typically, gating is used for contaminants that present high amplitude peaks in the time domain, including motion artifacts and the QRS complex of the ECG signal. The problem with the gating method is that it does not provide a continuous EMG signal, meaning that any event that occurs within the discarded time series will be completely ignored. A variant of this method is to set a threshold on the amplitude of the signal and to clip the amplitude to this value if the signal goes over the previously set threshold [30,31]. However, [27] established that the resulting square waves are more detrimental than the interference itself.

Since the aim of this paper is to review contaminant reduction methods that allow for the total reconstruction of the EMG signal, no further consideration will be given to this method.

### 3.3. Subtraction Methods in the Time Domain

**Figure 3:**
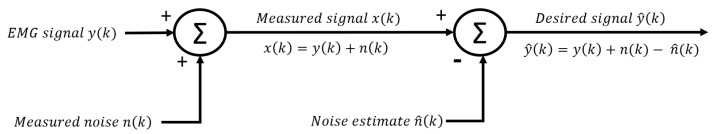
Principle of the subtraction methods in the time domain.

Subtraction methods can be employed to filter contaminants that present themselves as a finite number of waveform, such as PLI (with several of its harmonics) and ECG. The general idea is to estimate the characteristics of the waveform of the interference and to subtract this waveform directly from the signal in the time domain (Figure 3).

The mathematical explanation for this principle is as follows: if we can determine the characteristics of the noisy signal, we can define an estimate of the noise ($\hat{n}$). This allows us to find the estimated EMG signal ($\hat{y}$) using Equations (1) and (2):

(3) $$\hat{y}\left(t\right)=x\left(t\right)-\hat{n}\left(t\right)=y\left(t\right)+\left(n\left(t\right)-\hat{n}\left(t\right)\right).$$

Indeed, since $(n\left(t\right)-\hat{n}\left(t\right))\approx 0$, we obtain $\hat{y}\left(t\right)\approx y\left(t\right)$.

To this end, several schemes are possible. The methods presented below differ in the way that they estimate the waveform and in how they identify it in the signal.

#### 3.3.1. Estimation of PLI Using the Regression Method on a Reference Signal

As demonstrated by Fourier, any signal can be modeled as a superposition of sine waves. If we only consider the power-line, the noise component of the signal n(t) would therefore consist of a single sine wave whose frequency is 60 Hz or 50 Hz (depending on the geographical location). The definition of n(t) would then be

(4) n(t)=Asin(2πft+Φ),

where *A* is the amplitude of the signal, *f* is its frequency, and Φ represents the phase difference between the noise component n(t) and the measured signal x(t). Using trigonometric transformations, Equation (4) can also be written as follows:

(5) n(t)=A1sin(2πft)+A2cos(2πft).

Thus, if we can determine the characteristics of the noisy signal (*f*, *A* and Φ or *f*, A1 and A2 ), we can use Equation (3) to retrieve an estimate of the EMG signal ($\hat{y}$).

In 1998, ref. [17] developed a method based on this principle with the aim of reducing the power-line noise. The method, called “regression subtraction”, consists of performing two linear regressions on a reference signal that contains only the noisy signal ($x_{n}\left(t\right)$) to obtain the coefficients A1 and A2 from Equation (5). To do this, they posited that

(6) X1(t)=sin(2πft),

and

(7) X2(t)=cos(2πft),

Using these two equations, they stated the following two linear regressions:

(8) $$x_{n}\left(t\right)=a+bX1\left(t\right)+e1\left(t\right),$$

and

(9) $$x_{n}\left(t\right)=c+dX2\left(t\right)+e2\left(t\right),$$

where *a*, *b*, *c* and *d* are the coefficients of the regression, while e1 and e2 represent the error due to noise other than that of the power line contained in the signal. They calculate the coefficients of Equation (5): A1=b and A2=d and find the noiseless signal is estimated as follows:

(10) $$\hat{y}\left(t\right)=x\left(t\right)-\hat{n}\left(t\right)=x\left(t\right)-A1\sin\left(2\pi ft\right)-A2\cos\left(2\pi ft\right).$$

This procedure makes it possible to remove the PLI quite effectively; however, it can only reduce a single interference waveform. The authors in [17] therefore propose two variants to take into account the harmonics of the power line. The first is to perform the same workflow one by one with each of the harmonic frequencies. The other variation suggested is to use a higher order regression, such as a fifth-order polynomial.

#### 3.3.2. Estimation of PLI Using Spectral Analysis on a Reference Signal

Ref. [32] also employed a similar approach to attenuate the PLI. However, in contrast to [17], who estimated the coefficients using two regressions, they performed a frequency analysis on the reference noisy signal ($x_{n}\left(t\right)$) to obtain *A*, *f* and Φ. They also created the noisy waveform estimate using Equation (4) instead of Equation (5). Ref. [33] presented an analogous method using the stationary wavelet packet transform to estimate the amplitude and phase of the noisy signal. They also used two steps to refine the estimated coefficients. These two methods are a substantial improvement over regression-subtraction because they do not require prior information regarding the PLI frequency. Nonetheless, a major inconvenience of this idea is that it assumes that none of the PLI characteristics (*f*, *A*, and Φ) can change over time. Ref. [34] extended this approach by making it adaptive over the time of the trial. To do this, they used a signal model conceived of a set of harmonically related sine waves which are modulated by a polynomial equation. To estimate the polynomial coefficients, they perform several frequency analyses on windows of the signal over the trial. In this way, the amplitude and the frequency of each sine wave can be modulated over time according to the polynomial equation. They also extended their method to remove baseline wander (BW) by modelling it as a low-order polynomial.

#### 3.3.3. Estimation of PLI Using the Least Squares Algorithm on the Signal Itself

Using the same principle presented in Equation (3), we can define the mean squared error as follows:

(11) $$E=\frac{1}{M}\sum_{k=0}^{M-1}{\left(x\left(k\right)+n\left(k\right)-\hat{n}\left(k\right)\right)}^{2},$$

where *M* is the signal length. It can be shown that minimizing the mean square error *E* leads obligatorily to minimizing the error between *n* and $\hat{n}$. Indeed, since x(k) is the measured signal, it cannot vary. Therefore, any variation in *E* will result in a variation of $n\left(k\right)-\hat{n}\left(k\right)$. Ref. [35] used an iterative steepest descent to minimize E and to find the optimal characteristics of $\hat{n}$, assuming that PLI is defined as in Equation (5). To ensure the algorithm converges into an optimal minimum, they imposed a frequency range of 59.5 Hz to 60.5 Hz. As in the scheme presented by [17], their method assumes only one waveform. The advantage of their method over the earlier ones is that it does not require the use of a noisy signal reference. The algorithm can be applied directly on the sEMG signal.

#### 3.3.4. Template Estimation of the ECG Interference Signal

Estimating PLI comes down to simply finding the coefficients of Equation (2) (or Equation (4)). However, in some cases, it is not possible to model the interference signal as a simple sine (or cosine) wave. In these cases, a template of the interference signal must be estimated. For example, the ECG signal cannot be modelled easily. A template of the ECG signal must therefore be defined. The ECG template subtraction works under the hypothesis that the ECG signal is quasi-periodic. It involves the subtraction of an ECG template from the EMG signal at each occurrence of an ECG waveform. This means the method can be separated into three steps: 1. template estimation, 2. detection of the QRS complex and 3. subtraction of the template. However, these steps can be embedded in one another. For example, template estimation often includes detection of the interference waveforms. Moreover, in some cases, steps 2 and 3 can be performed together and the template can also be scaled. Ref. [36] proposed a method for estimating this template by first identifying the ECG peaks (R waves) using a threshold on the signal’s amplitude and then, averaging the R waves. This second step is performed using the raw signal x(t) (EMG + ECG). However, by aligning the R waves and using a long enough signal, the EMG signal will tend to be removed through the averaging process. Since the ECG signal is non-stationary and may vary in amplitude and phase, Ref. [36] used a least squares algorithm to identify and scale the ECG template according to the raw signal. The subtraction is then performed. Ref. [37] reported a similar procedure in which multiple different templates can be created. An operator can then choose the best template to be applied at each occurrence of the QRS complex. Ref. [38] introduced a simplified solution in which they estimate an ECG template to be subtracted from the diaphragmatic EMG signal by considering only the expiration phase, during which only the ECG signal is present. Using cross-correlation, the previously selected ECG waveforms are aligned and averaged to create the template. In the same way, a correlation between the entire signal and the template is used to trace the ECG waveform and subtract the template from the signal.

More recently, ref. [39] submitted a distinct approach in which two different moving averages are applied to the rectified signal in order to detect the QRS complex. The intersection of the two resulting filtered signals is used as the peak detection. As in the other methods, the ECG waveforms are averaged to estimate the template. Ref. [40] proposed an improved version of this method by making it adjustable to the amplitude variations. A comparable approach was put forward by [41] that uses the median instead of the average of the ECG waveforms to obtain the template. This work also presents a scheme to adjust the template so that it fits each actual ECG wave duration.

The main disadvantage of all the template subtraction methods presented so far is that they assume the ECG artifact has a constant shape across the entire trial. In some of the cases presented, while the amplitude or the duration of the template can be modulated, the shape of the template remains unchanged. Moreover, to perform the average/median, we also make the supposition that the shape does not change.

#### 3.3.5. Adaptive Estimation of the Interference Signal by Means of Filtering the Raw Signal

**Figure 4:**
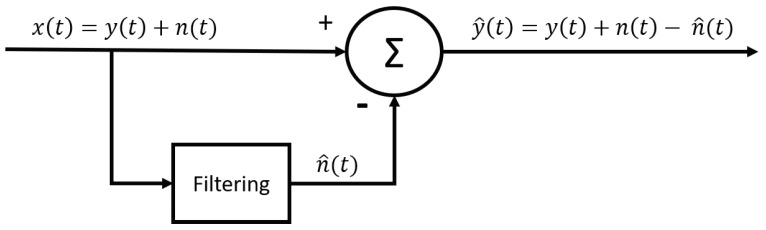
General block diagram of an interference reduction method using an adaptive estimation of the interference signal by means of filtering the raw signal.

Instead of using filters to remove interference from the EMG signal, this method employs filters to retrieve only the interference signal $\hat{n}$. After this, the interference signal can be subtracted from the raw signal to obtain the EMG signal (Figure 4). This allows an adaptive estimation of the interference to be obtained over the trial. The shape, amplitude and duration of the interference is adjusted over the trial according to the raw signal.

Ref. [42] proposed using a moving average and a moving median to extract the motion artifact from the EMG signal. In their workflow, they substitute the moving average/median calculated on each window to the central point of the window. The obtained signal is called the trace and is considered the artifact’s signal. The trace is then subtracted from the raw signal to obtain the uncontaminated signal. The researchers concluded that the moving median is more effective at removing the MA while preserving the EMG signal when compared to the moving average technique and a high-pass filter with a 50 Hz cutoff frequency. However, in a subsequent paper, this method was also compared to one based on the wavelet transform and was found to be inferior [43].

A similar scheme was introduced by [44] to extract the ECG signal. Their work uses two moving averages in order to adequately estimate the high- and low-frequency parts of the ECG signal. They then use a derivative threshold algorithm to detect the QRS complex and to determine which part of the signal is better characterised by either the high- or low-frequency estimation of the wave. Ref. [45] proposed another solution in which only the QRS complexes of the ECG signal are removed. A zero-phase-shift low-pass filter with a 30 Hz cutoff frequency is used to estimate the ECG signal. The time intervals containing the QRS complexes are identified and the estimated ECG interference is then subtracted only during these intervals, making it useless for estimating the lower frequency parts of the ECG waveform. However, a limitation of this method is that they use an ECG reference signal to detect the QRS time intervals, which means a supplementary channel is needed. Another limitation of this method is the assumption that the low-frequency components of the EMG signal (<30 Hz) and the high-frequency components of the ECG artifact (>30 Hz) are both negligible. Indeed, the low-pass filtered signal is thought to contain only the ECG interference, and in the same way, any ECG signal above the cutoff frequency will remain in the signal. Moreover, the ECG interference between QRS complexes is not removed either. Yet, the advantage of these last two methods over a simple high-pass filter is that the detection of the QRS complex allows users to either change the filter’s cutoff frequency [44] or totally ignore the filter when it is not necessary [45]. This helps to preserve the EMG signal as much as possible when the high amplitude parts of the ECG signal are not present.

In the same vein, inspired by previous work focused on removing PLI from the ECG signal, ref. [26] presented a method in which the PLI is not modelled as a sine (or cosine) wave. Instead, an adaptive estimation of the power-line interference is generated by applying band-pass filters (49.5 Hz to 50.5 Hz and 149.5 to 150.5 Hz) directly on the raw signal in order to estimate the interference signal and be able to remove it from the original EMG signal. However, using the least mean squares (LMS) algorithm for both methods, they compared it to the method that assumes a sine/cosine wave and concluded that the sine/cosine modelling of the PLI was more effective.

#### 3.3.6. Adaptive Noise Canceller (ANC)

**Figure 5:**
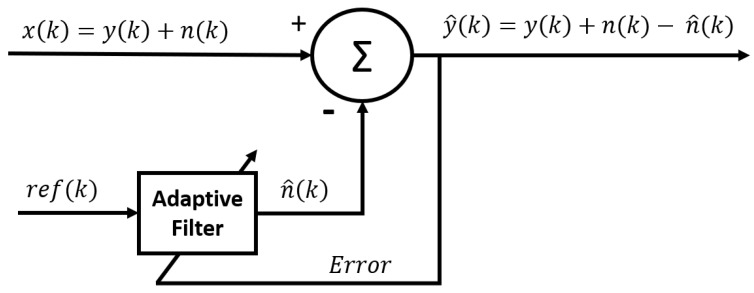
Block diagram of the classic adaptive noise canceller.

One of the main difficulties with the methods presented above is that, in addition to having to determine the frequency and the amplitude of the signal that we wish to reject, it is also necessary to determine its phase with respect to the measured signal. Indeed, if we remove a sinusoidal signal containing a phase shift with the real noise, we risk increasing the noise instead of reducing it. Another way to remove the interference signal is to use an adaptive noise (or interference) canceller [46]. This technique employs a noise reference signal, ref(k), that is acquired at the same time as the EMG signal via another channel. For example, another sensor could be used to acquire the ECG signal at the same time as the EMG signal in order to cancel the ECG artifact. Contrary to what one might think, the interference signal is not directly subtracted from the raw signal. Indeed, the amplitude of the noise can be different in the measured signal than in the reference. Further, as these signals are not acquired at the exact same location, there may be a phase delay between the noise measured with the EMG signal and the reference. Therefore, the reference signal is modified using an adaptive algorithm in order to estimate the noise $\hat{n}$. Then, as in the previously presented methods, $\hat{n}$ is subtracted from the raw signal x(k) (Figure 5).

In the classic adaptive noise canceller (ANC), the noise estimation ($\hat{n}$) is obtained by applying an adaptive finite impulse response (FIR) filter to the reference signal:

(12) $$\hat{n}\left(k\right)=\sum_{i=0}^{N-1}w_{i}\left(k\right)\cdot ref\left(k-i\right),$$

where *N* is the order of the filter and $w_{i}\left(k\right)$ is the *i*th weight of the FIR filter for the *k*th sample. As mentioned earlier, the FIR filter is adaptive. This means the weights are adjusted over time so that $\hat{n}\left(k\right)$ is as close as possible to n(k). The weights are updated using a minimization algorithm. As presented in a previous method, it can be shown that minimizing the mean square error of the output ($E(\hat{y}\left(k\right))$) leads to the minimization of the error between *n* and $\hat{n}$. Indeed, since y(k) is the measured EMG signal, it cannot vary. Therefore, any variation of ($E(\hat{y}\left(k\right))$) will result in a variation of $n\left(k\right)-\hat{n}\left(k\right)$. This is why $E(\hat{y}\left(k\right))$ is used as an estimation of the error. The most common algorithm used to adapt the weights is the least mean square (LMS) algorithm. The update is performed as presented in the following equation:

(13) $$W\left(k+1\right)=W\left(k\right)+2\cdot \mu \cdot \hat{y}\left(k\right)\cdot R\left(k\right),$$

where W(k) is a vector of $w_{i}\left(k\right)$ at sample *k*, and R(k) is the reference input signal vector that goes from ref(k) to ref(k−(N−1)). The parameter μ is called the step size and is responsible for the performance of the cancelling process (stability and convergence rate). Ref. [47] presented a method based on the ANC using a FIR filter (N=10) and an LMS algorithm to eliminate the ECG interference from the EMG signal. Since then, many variations of this method have been reported [26,48,49,50]. For example, ref. [48] presented a similar scheme in which the step size μ is adjusted for the different input samples in order to maximize the convergence rate. Other adaptive algorithms have also been proposed. The second most known adaptive filter algorithm in the literature is the recursive least square (RLS). The main difference between the two algorithms is that RLS adapts the weights based on the total error computed from the beginning while the LMS adapts the weights according only to the error on the last sample. The RLS algorithm was used by [51] and a variant of the RLS algorithm was also submitted by [52] to remove the ECG artifact.

The classic ANC based on the FIR filter and the LMS algorithm were also employed by [53] to remove the power-line interference. In this case, the reference signal (ref(k)) is modelled by a cosine wave. This means that, in contrast to the ANC used to remove the ECG interference, the reference is constructed mathematically. Compared with the other PLI removal methods reported previously, the difference here is that PLI is modelled as a single cosine wave with a random amplitude. Instead of using a second wave (sine), as in Equation (5), or even iterating to find the coefficient of the phase from Equation (4), the phase difference between the PLI reference and the signal is accounted for using an adaptive FIR filter, as presented in Equation (12). The idea behind this is to use a filter order that allows for the consideration of enough points from the reference to have a complete sine to account for any phase difference. For example, if we have zeros for all weights except the middle weight, the resulting $\hat{n}$ would be a sine with an amplitude corresponding to the value of the middle weight. Using this filter, it is thus possible to optimize the weights in order to minimize the difference between the measured signal and $\hat{n}$, as for any ANC. Ref. [54] presented a derived version of this procedure, adapting it to also take into account the harmonics of PLI. Likewise, ref. [55] designed an ANC by replacing the FIR filter with a Laguerre filter. In their workflow, each section of the Laguerre filter changes the phase of the reference signal. Accordingly, an estimate of the PLI signal can be computed as the weighted linear combination of the outputs of the filters.

#### 3.3.7. Nonlinear ANC

**Figure 6:**
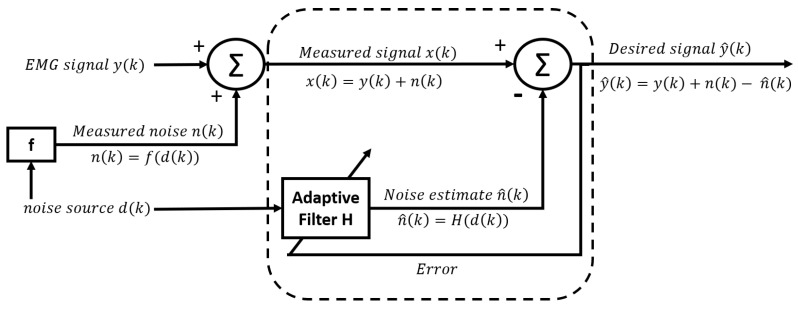
Block diagram of the adaptive noise canceller along with the measured signal composition.

In all of the methods presented above, it is assumed that the waveform of the interference can be approximated using a linear combination of the successive weighted samples of the reference signal. Therefore, referring to Figure 6, f(d(k)) has always been considered a linear function.

The adaptive filter H(d(k)) used to estimate this function was thus also linear. However, it is defended that the superimposition of the EMG and the ECG signal exhibit some nonlinear and time-variant distortions [56]. Therefore, it has been suggested that the concept of the linear ANC could be extended by using nonlinear adaptive filters (*H*). Among them, the back propagation network (BPN), cascade correlation network (CCN) and adaptive neuro fuzzy inference system (ANFIS) were investigated [57,58]. An event-synchronous adaptive interference canceller (ESAIC) was also considered to remove the ECG interference. However, its redundancy was criticized by [56], who designed a variant of the ESAIC (ESC) that overcomes this problem. Other nonlinear ANC using the state space were also investigated [59,60].

### 3.4. Denoising Methods after Signal Decomposition

**Figure 7:**
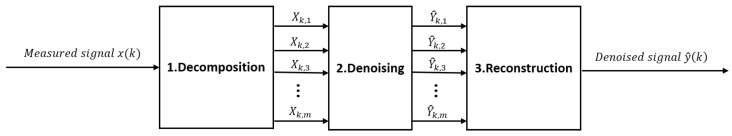
Block diagram of the decomposition methods.

In all the methods presented until now, the denoising had to be performed one waveform at a time. However, some contaminants such as WGN do not present themselves as a finite number of waveforms. Therefore, subtraction methods in the time domain are not suitable in these cases. One solution that has been proposed to overcome this problem consists of decomposing the signal in the frequency domain (or other) and to perform the denoising on the coefficients of the decomposition rather than on the time domain signal. The method therefore includes three steps: decomposition, denoising and reconstruction of the signal in the time domain (Figure 7).

Many methods have been proposed based on this principle. The methods differ primarily in the decomposition algorithm but also in the method used to denoise the decomposed signal. Therefore, a multitude of variants of this approach will be described below.

#### 3.4.1. Decomposition Methods after Fourier Decomposition

In the 1940s, the American mathematician Nobert Wiener introduced a new filter called the Wiener filter [46]. This filter consists of applying a Fourier transform to the signal. The obtained Fourier coefficients are then modified according to the ratio between the expected signal spectrum and the actual spectrum. According to this definition, conventionally, the desired signal is known. However, the desired EMG signal is generally unknown. Several variants of the Wiener filter have therefore been proposed to filter the EMG signal. Among other things, most of these variants consist of using an estimate of the noisy signal as a reference rather than the desired signal.

**Figure 8:**
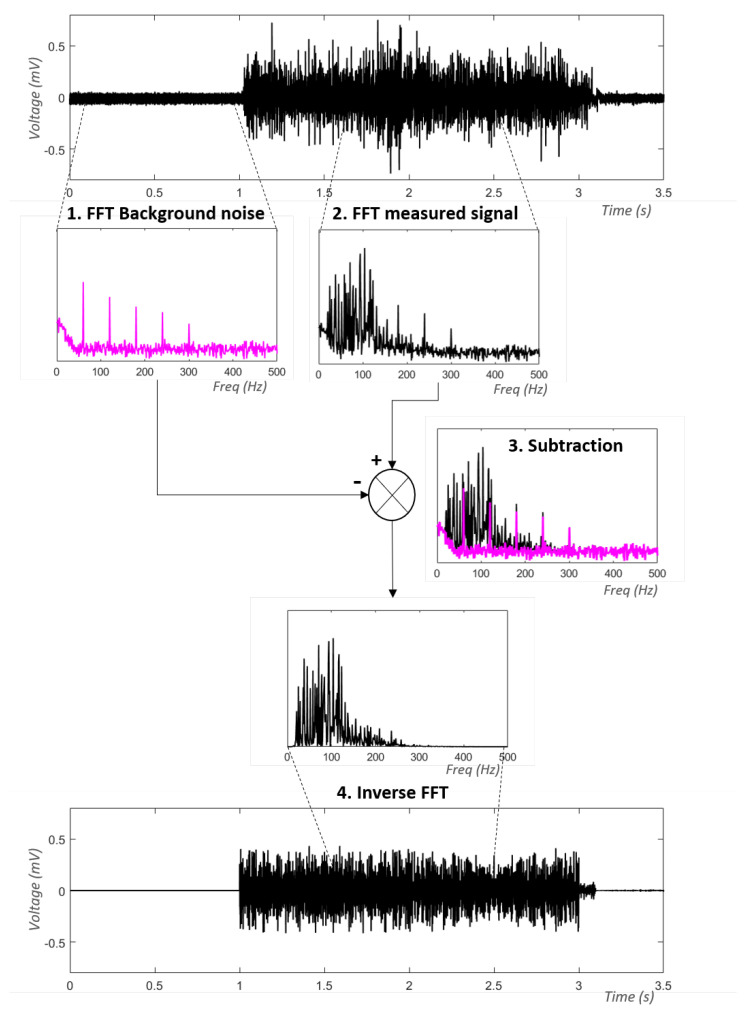
General scheme of the method proposed by [17] to remove background noise from the EMG signal: 1. Estimation of the power spectrum coefficients of the Background noise by performing a fast Fourier transform (FFT) on the noisy signal (the electrode is placed on the skin, but the muscle is not contracted), 2. Estimation of the power spectrum coefficients of the measured signal during contraction using the FFT, 3. Subtraction of the noise coefficients from the measured coefficients and 4. Reconstruction of the signal using the inverse Fourier Transform.

In 1998, ref. [17] presented an approach using the Fourier transform to estimate the power spectrum of the noisy signal from a reference signal obtained at the start of the trial. During this period, the electrode is placed on the skin, but the muscle is not contracted. We therefore obtain an estimate of the background noise. This solution makes it possible to consider several noise sources without having to identify them. Unlike time domain subtraction methods, the reduction of noisy waves at several different frequencies is therefore quite simple. To reduce the noise, the signal of interest is therefore also decomposed in order to obtain its power spectrum. Then, the coefficients of the noisy signal are subtracted from those of the measured signal. In this way, an estimate of the power spectrum of the non-noisy signal is obtained. The general scheme of this method is depicted in Figure 8.

Ref. [61] also reproduced this procedure to reduce the contamination caused by electrical stimulation. Unlike [17], this approach directly uses coefficients of the Fourier transform rather than the power spectrum. In addition, to estimate the coefficients of the noisy signal, the researchers developed a more robust method that involves performing several consecutive Fourier transforms and averaging over the coefficients obtained at different times. This frequency subtraction method was also tested by [12] to remove the ECG from the EMG signal. However, after comparing its performance to other ECG removal methods, they concluded that it was not the strongest method. Ref. [62] designed a comparable solution in which the background noise is acquired in real time via a separated channel. Similar to the ANC, this therefore allows them to obtain an estimate of the instantaneous noise rather than using an estimate based on a signal taken at the start of a trial. This method differs from the ANC in that the denoising is performed in the frequency domain rather than the time domain. Another analogous method that uses a dual-adapted FBLSM was introduced by [63]. Their method employs an adaptive finite impulse response (FIR) filter in the frequency domain.

An algorithm based on the discrete cosine transform (DCT), which is derived from the Fourier transform, was also explored by [64]. They removed the PLI by setting to zero the coefficients of the DCT corresponding to the PLI. However, a drawback of this method is that, like the conventional notch filter, it also removes a major part of the EMG signal at this frequency. Ref. [65] have explored an alternative to this method, called the spectrum interpolation, in which the spike in the signal’s power spectrum caused by PLI is first removed. The real spectrum value at 60/50 Hz is estimated by interpolating between the next two values in the spectrum. The signal is then reconstructed using the modified coefficients. This procedure allows for the attenuation of the PLI without removing the EMG signal at 60/50 Hz, which is a major accomplishment compared to most methods. A variant of this scheme was also introduced by [66] who added an Hampel filter in order to detect the spikes in the power spectrum. In the original method presented by [65], the center frequency had to be known for the method to be automatically performed. However, in some cases, PLI harmonics may also be present in the signal. In addition, the center frequency of the PLI can also vary slightly. The method proposed by [66] thus allows more than one spike to be detected at any frequency. This also opens the door to the removal of other interference such as electrical stimulation.

#### 3.4.2. Denoising Methods after Wavelet Decomposition

Wavelet transform (WT) is a decomposition method created as an alternative to the Fourier transform. The method is analogous to the those already presented in the following ways: 1. The signal is decomposed using one of the wavelet transforms, 2. It is then denoised in the wavelet domain and 3. The signal is subsequently reconstructed by applying the inverse wavelet transform on the modified coefficients. In general, wavelet-based denoising methods are used to reduce the WGN. While the general block diagram is similar to the methods based on the Fourier transform and cosine transform, the decomposition using the wavelet transform is not as trivial, and many parameters must be defined. Therefore, much more research has been conducted on the subject. Moreover, a considerable amount of research has focused on wavelet denoising in the context of the WGN removal. Therefore, in most cases, the denoising stage differs from that of the previously mentioned decomposition methods.

**Step 1. Decomposition using wavelet transform**: While the Fourier transform decomposes the signal from the time domain into the frequency domain, the wavelet transform generates components (kernels) that are defined in terms of both time and frequency. In this approach, the components are created by translating and dilating a fixed function called the mother wavelet (Ψ(t), thus allowing a multi-dimensional representation of the signal. Unlike the sine/cosine waves used in the Fourier transform, the amplitude of the mother wavelet varies across its length. Translating it over the signal thus allows the definition of each component in time.

**Figure 9:**
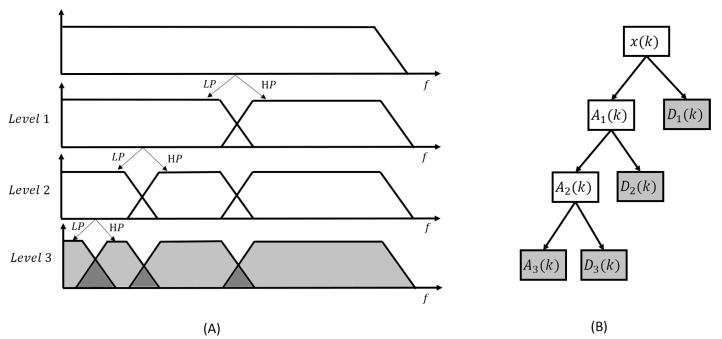
(**A**) Filter bank resulting from a a DWT at level 3 of decomposition and (**B**) the resulting coefficients of the DWT. The coefficients/components used in the DWT are presented in grey.

Wavelet transforms can be categorized into two main types: continuous wavelet transform (CWT) and discrete wavelet transform (DWT). CWT involves calculating the wavelet coefficients at every possible scale. However, this version of WT is highly redundant and computationally time consuming. From a denoising perspective, the aim is to decompose the signal in a way that allows for the reconstruction of the original signal using a linear combination of the smallest number of components. In the classic CWT, many more coefficients are generated than are actually needed to reconstruct the signal. CWT is thus highly redundant. As stated by [67], the wavelet functions must be orthogonal in order to meet this criteria. DWT achieves this by restricting the variation in translation and scale to powers of 2. As presented in Figure 9, DWT can be implemented using Mallat’s algorithm, which uses high- and low-pass filters to separate high- (D) and low-frequency (A) components [68].

However, in contrast to the Fourier transform, the resolution of wavelet transform is not uniform in the time–frequency plane [68]. Indeed, as stated in the Heisenberg problem, larger scales cover larger time frames but smaller frequency frames, while smaller scales expand the frames in frequency and contract them in time. Therefore, at each level of decomposition in the classic DWT, the low-frequency component is decomposed into two new components using a high-pass filter. The filter bank resulting from a level 3 DWT is presented in Figure 9A. The coefficients obtained using the level 3 DWT on a measured signal (x(k)) are presented in Figure 9B. The measured signal can then be rewritten in the wavelet domain as a linear combination of the high-frequency coefficients ($D_{j}\left(k\right)$) and the last low-frequency coefficient ($A_{L}\left(k\right)$), as follows:

(14) $$X\left(k\right)=A_{L}\left(k\right)\sum_{j=1}^{L}D_{j}\left(k\right),$$

where *L* is the level of decomposition.

**Figure 10:**
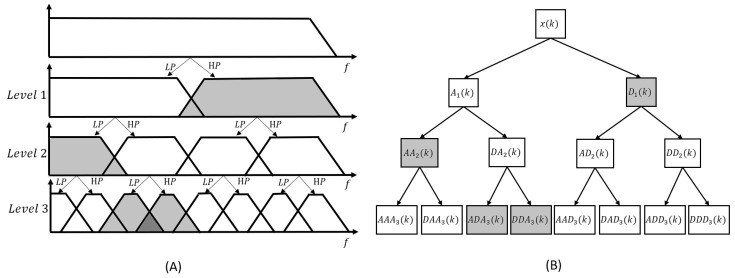
(**A**) Resulting filter bank of a WPT at level 3 of decomposition along with (**B**) resulting coefficients of the WPT. The coefficients/components used in WPT are presented in grey.

A variant of DWT is the wavelet packet transform (WPT) [69]. The main difference between the two methods is that WPT is more adaptive to the signal. As shown in Figure 10, it decomposes not only low-frequency components, but also high-frequency components at each level. Using the signal itself, the most useful frequency bands can be selected to match the signal. The signal can then be expressed as any orthogonal combination of components, as shown in grey in Figure 10.

Another variant of the DWT is the stationary wavelet transform (SWT). This variant was designed to overcome the variance DWT suffers due to time shifts [70]. SWT is similar to DWT, except that at each level of decomposition, the filters are up-sampled and the signal is never sub-sampled.

In addition to choosing the WT type, the mother wavelet and the level of decomposition must also be defined. According to the literature, it seems that the best mother wavelet has yet to be clearly defined. Many different mother wavelets have been reported in EMG signal denoising. Likewise, the decomposition level also seems to differ across the literature. However, a number of studies have compared mother wavelets and decomposition levels in the context of EMG signal denoising. Ref. [71] tested a total of 53 mother wavelets from the following famillies: Daubechies, Symlets, Coiflet, BiorSplines, ReverseBior and Discrete Meyer. Their results suggest that the most adapted wavelets in the context of removing WGN from EMG signals are first-order Daubechies, BioSplines, and ReverseBior wavelets (db1, bior1.1 and rbio1.1). They also found that various mother wavelets produce relatively good results. However, they did not recommend bior3.1, bior3.3, bior3.5, bior3.7, bior3.9 and dmey. Ref. [72] also investigated the performance of different mother wavelets with two different decomposition levels. They reported better results using the db4 at level 3. These results appear to be consistent with the literature, since most studies have reported using either the db2 [73,74,75,76,77,78,79] or the db4 [67,80,81,82,83,84]. In terms of the optimal decomposition level, level 4 appears to be the most widely used [5,19,71,77,82,85,86], while the level 3 is also frequently chosen [25,72,87,88,89,90].

**Step 2. Denoising in the wavelet domain:** As for all decomposition methods, once decomposition is completed, the signal is denoised in the decomposition domain. While the block diagram of wavelet denoising methods is similar to those based on the Fourier transform and cosine transform, a considerable amount of research has focused on wavelet denoising in the context of WGN removal. Therefore, instead of simply subtracting the coefficients obtained with the noisy signal from those of the trial, the denoising stage is usually performed by applying a threshold to each of the coefficients, as proposed by [91]. Thus, this stage is separated into two main steps: 1. threshold selection and 2. application of the thresholds.

Threshold selection: The threshold selection step is used to determine the threshold value that should be applied to each coefficient of the decomposed signal. There are different algorithms that help to define these thresholds. When WGN is the only noise source considered, Equation (1) becomes

(15) x(t)=y(t)+ση(t)

where η is Gaussian noise N (0, 1) and σ is an estimate of the noise variance.

In the wavelet domain, this equation becomes

(16) $$X_{i,m}=Y_{i,m}+\sigma N_{i,m}.$$

In WGN removal, the principle is that the noise level (σ) can be estimated using the signal itself. In the classic method submitted by [91], the Universal Threshold is defined, as follows:

(17) $$THR_{UNI}=\hat{\sigma }\sqrt{2log(M)},$$

where $THR_{UNI}$ is the threshold, *M* is the signal length in the time domain and $\hat{\sigma }$ is the noise estimate. The noise estimate $\hat{\sigma }$ is usually obtained using Equation (18).

(18) $$\hat{\sigma }=\frac{median(D_{j})}{0.6745},$$

where $D_{j}$ is the detail coefficient at level *j*, which means that a noise estimate is obtained for each coefficient. This noise estimate is referred to as level dependant. However, two other noise estimates are also used frequently in wavelet denoising [71]. The noise level can also be estimated using only the first level. In fact, it is well known that close to no EMG signal can be found in the first detail coefficient $D_{1}$. Therefore, this coefficient is often used as an estimate of the overall noise. Since the noise is assumed to be WGN, the noise is equally dispersed over all frequencies, which means that it can easily be estimated from a single coefficient. The last method is said to be global and defines $\hat{\sigma }$ as an estimate of the standard deviation of all the wavelet coefficients [82].

Since [91] published their method, several other threshold selection procedures have also been explored. The most popular are the SURE Threshold, Hybrid Threshold and Minimax Threshold [73].

Application of the thresholds: Once they have been defined, thresholds are used to either remove or shrink the coefficients according to a threshold function. The simplest threshold function is the hard function (HAD), which consists of zeroing all coefficients below their associated thresholds. The HAD function can be stated as follows:

(19) $$D_{j}=\left\{\begin{array}{cc} D_{j} & \mathrm{if}\;D_{j}>THR_{j}\;\\ 0 & \mathrm{otherwise} \end{array}\right.$$

Another popular function is the soft function (SOF), which is an extension of the hard function [92]. In addition to zeroing the coefficients at values lower than their corresponding thresholds, the other coefficients are also shrunk by subtracting the threshold values from them:

(20) $$D_{j}=\left\{\begin{array}{cc} sgn\left(D_{j}\right)\left|D_{j}-THR_{j}\right| & \mathrm{if}\;D_{j}>THR_{j}\;\\ 0 & \mathrm{otherwise} \end{array}\right.$$

**Figure 11:**
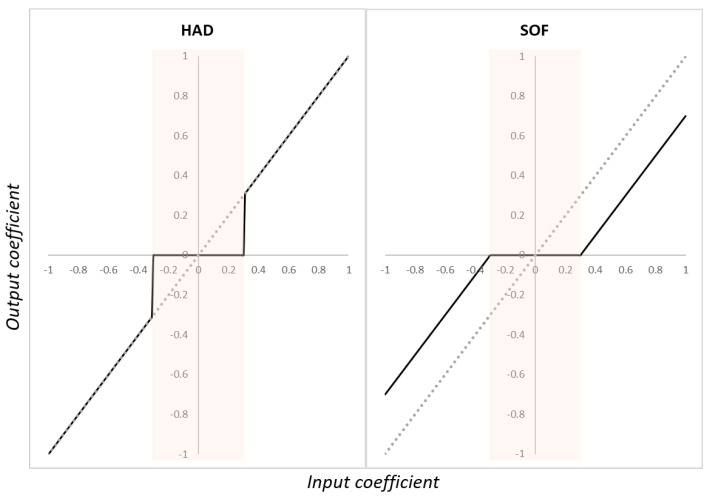
Output coefficient obtained according to the input coefficient for HAD (**left**) and SOF (**right**) functions.

A comparison of the output coefficient obtained according to the input coefficient is presented in Figure 11 for both the HAD and SOF functions. In addition, other functions such as the hyperbolic and non-negative garrote have also been investigated [75].

Aside from the classic wavelet thresholding method, which consists of applying a threshold to the coefficients, other denoising methods using the wavelet have been reported in the literature. Ref. [93] designed an algorithm based on the WT to reduce ECG artifacts in the signal. Since the ECG signal tends to have the most energy in the combined EMG + ECG signals, these researchers suggest completely removing the largest coefficients. Using this approach, their thresholding function is the complete opposite of the classic HAD function:

(21) $$D_{j}=\left\{\begin{array}{cc} 0 & \mathrm{if}\;D_{j}>THR_{j}\;\\ D_{j} & \mathrm{otherwise} \end{array}\right.$$

Other studies in which contaminants other than WGN have been considered suggest choosing the thresholds manually. For example, ref. [53] uses a manual thresholding method to remove motion artifacts. Similarly, ref. [94] applies the manual thresholding to the removal of unscaled white noise.

Aside from this, ref. [74] proposed a method in which only the lower frequency component ($A_{L}$) is ignored in the reconstruction. This allowed the researchers to eliminate the baseline wander, which, by definition, is found in the lower frequencies. In contrast, ref. [67] presented a comparable approach in which only the lower components are used in the reconstruction. It is well known that the ratio of EMG signal versus noise in high-frequency components is low. Therefore, the researchers suggest ignoring the higher frequency components in the reconstruction. Ref. [77] proposed using a quiet trial to estimate the background noise as reported with the Fourier transform. Using this estimation, they were able to set the thresholds accordingly. A comparable scheme to remove background noise was also reported by [70].

**Step 3. Reconstruction using the modified coefficients**: The modified coefficients are then used to reconstruct the signal in the time domain using the inverse wavelet transform (IWT).

#### 3.4.3. Denoising after Empirical Mode Decomposition (EMD)

In 1998, ref. [95] developed a new decomposition method called the empirical-mode decomposition. Unlike the wavelet approach, EMD does not decompose the signal in terms of basic atoms like mother wavelets, but rather breaks them into a series of intrinsic mode functions (IMFs). These functions are obtained using a procedure called the sifting process. The biggest difference between WT and EMD is that EMD adapts to the signal that needs to be decomposed. As explained previously, with WT, the mother wavelet and the level of decomposition must be defined. This results in a certain number of filters that are fixed. In contrast, with EMD, the IMFs are not fixed and depend on the signal. This allows a better adaptation of the decomposition according to the input signal.

In 2006, ref. [7] used EMD to filter WGN from an EMG signal. Similar to Donoho’s wavelet method, the approach consists of applying a threshold to the coefficients obtained from the decomposition into IMFs and reconstructing the signal with the modified coefficients. However, in this study, the selection of the thresholds was conducted differently. They performed an EMD on a noise window taken before the trial. The coefficients obtained with the noisy signal were used as thresholds for the trial by applying the SOF function. EMD-based denoising methods have also been performed to remove electrical stimulation [96] and ECG [97]. Ref. [98] developed a novel denoising algorithm inspired by wavelet thresholding called interval thresholding (IT), which is performed on the IMFs obtained from the EMD. Other thresholding methods for EMD denoising were also studied by [99]. Their findings suggest that an iterative version of the IT yields better denoising results when employed with the hard threshold.

Subsequently, ref. [100] used an extension of EMD called the ensemble EMD (EEMD) developed by [101]. Without going into detail, in EEMD, noise is added to the signal purposefully in order to improve the decomposition into IMFs. To do this, several EMDs are performed one after the other so that the results can be averaged. The results of this study demonstrated that the EEMD was more efficient in reducing the WGN, PLI and BW than conventional filters and classic EMD, particularly for low SNR. Another denoising algorithm based on EEMD and an improved wavelet threshold was introduced by [102] to remove random noise.

An improved algorithm derived from the EEMD was also introduced by [103]. This Complementary EEMD (CEEMD) was designed to reduce the contamination introduced by the added noise. In CEEMD, each noise is added in pairs with plus and minus signs to insure a complete cancellation of the residual noise in the reconstruction step. CEEMD was applied to EMG denoising by [104] along with an improved version of the interval thresholding (IT) method designed by [98]. They compared the results obtained with their proposed method (CEEMD-IT) to those of SWT, EMD alone, EMD with IT and EMD with direct thresholding in the context of the WGN removal. The study demonstrated that the CEEMD-IT method achieved better denoising results for most of the tested SNR while retaining more useful information.

A further extension of the EMD method, called BoostEMD, was designed by [105]. This variant of the EMD method allows for the use of higher order IMFs. The authors conducted a comparative study with the classic EMD for classification accuracy and found that their method was more effective. However, they also observe that the nature of their method makes it impossible to extract all the noise while retaining all the useful information in the EMG signal.

#### 3.4.4. Denoising after Variational Mode Decomposition (VMD)

Although EMD has many advantages, it also has disadvantages, including its sensitivity to noise and the fact that the frequency bands of the components may overlap each other [106]. To address issues of the EMD method, ref. [107] have developed a new decomposition method called variational mode decomposition (VMD). The VMD method is in fact an extension of the Wiener filter applied over several frequency bands. It decomposes the signal into variational mode functions (VMFs), each of which has its own center frequency and narrow band. The fact that the VMFs are updated constantly makes the method adaptive.

Two EMG denoising schemes based on VMD were developed by [106]. The first is performed with soft thresholding, while the second is performed with soft interval thresholding (SIT). Since the VMFs and their respective center frequency can be obtained from the model, the signal is reconstructed using the inverse Fourier transform. Ref. [106] compared their VMD-based methods to both the EMD-based and wavelet-based methods in terms of SNR and RMSE and demonstrated that both proposed methods performed better at denoising. Moreover, they showed that the VMD-SIT version was more effective than the VMD-WST, in addition to producing a smoother and continuous signal. Ref. [108] also performed a comparative study of the VMD, EMD, EEMD and conventional IIR filters methods to remove the WGN, PLI and baseline wander. They demonstrated that the VMD-based method performed better for removing the WGN and BW, and that it was also effective in removing the PLI when the SNR was low.

### 3.5. Combining Methods and Hybrid Methods

#### 3.5.1. Wavelet-ICA and EMD-ICA

**Figure 12:**
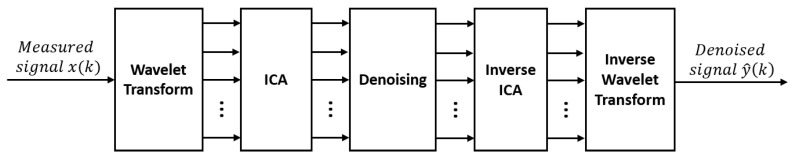
Block diagram of Wavelet-ICA.

In the denoising of multichannel EMG signals, a common approach is to use a blind source separation (BSS) technique, such as an independent component analysis (ICA), Canonical correlation analysis (CCA), or principal component analysis (PCA) to extract statistically independent components from a set of measured signals [109]. The idea behind using one of these algorithms is to isolate the source of the interference and remove it from the signal. However, these algorithms are only suitable for use in multichannel denoising, since they need more than one measured signal to be able to recognize the contribution of each source. In 2004, ref. [110] introduced a method combining the wavelet transform and ICA to denoise the multichannel EMG. This combined method was adopted by [111,112,113] to remove ECG from single-channel EMG signals. Indeed, to successfully use a BSS technique, more than one measured signal is needed. However, this combined method relies on the initial decomposition of the single channel signal using the wavelet transform, thus producing a multidimensional signal. ICA is then performed directly on the WT output to separate the interference signal from the EMG signal. As presented in Figure 12, denoising is performed after ICA on the sources that are considered interference. The signal is then reconstructed using inverse ICA followed by inverse WT. In contrast to the classic wavelet denoising methods presented earlier, the decomposition level used here appears to be higher. In fact, the authors used 6 [111], 8 [113] and 18 [112] levels of decomposition.

Similarly, ref. [114] presented a method combining the EEMD with ICA to separate ECG from EMG signals. In their paper, they also propose a single channel ICA (SCICA), in which the original signal is broken up into several signals of the same length. A mutlichannel signal is thus simulated by these truncated signals. However, ref. [114] compared the latter to their EEMD-ICA method along with the wavelet-ICA (WT-ICA) method and found out that SCICA had the worst performance. They concluded that WT-ICA and EMD-ICA presented a comparable performance on real signals, while WT-ICA showed a weaker performance on simulated signals.

#### 3.5.2. Wavelet-Adaptive

**Figure 13:**
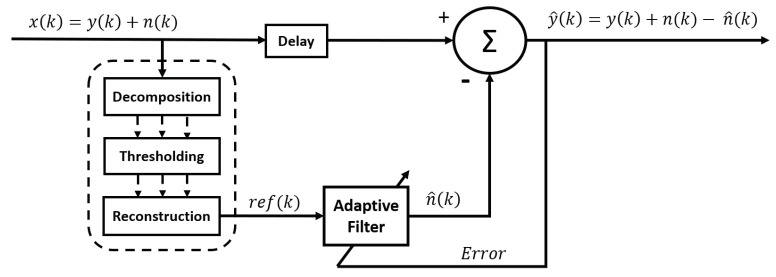
Block diagram of adaptive filtering using wavelet transform on the raw signal to estimate the interference.

A variant of the adaptive noise canceller was also submitted by [81] to remove the ECG interference from the EMG signal without using an extra channel to record the ECG reference. In contrast to the classic ANC, the ECG reference signal is obtained directly from the measured signal by performing WT (Figure 13). In the wavelet domain, the coefficients are thresholded so that they retain only the ECG part of the signal. The reconstructed signal is used as reference for the ANC. This method was also reproduced by [115] to get rid of MA.

#### 3.5.3. Wavelet-Wiener and FFT-Wiener

Ref. [5] have developed a method based on the wavelet transform. Instead of using the thresholding methods presented above, the denoising is performed using a wiener filter. As mentioned before, the wiener filter needs an a priori estimate of the desired EMG signal. In their method, this estimation is obtained using a second WT. The coefficients of the second WT are thresholded so that they retain only the noise-free signal. The reconstructed signal is used as a reference for the wiener filter. Ref. [116] presented a similar scheme using the Fourier transform.

## 4. Performance Evaluation

The contamination reduction method is always a compromise between removing the contaminants and preserving the EMG information [10]. Thus, in additon to assessing the method’s ability to remove contaminants, we also need to verify that it does not have a large negative effect on the EMG signal. The goal is to filter the maximum amount of noise while retaining as much of the desired EMG signal frequency spectrum as possible. The second issue, aside from reducing contamination, is to achieve minimal distortion, which means that the relative contribution of any frequency component in the EMG signal should not be altered [29].

The performance of a contamination reduction method is evaluated by performing it on real or simulated EMG signals with real or simulated contaminants.

Using simulated signals allows for a comparison of the denoised signal to a known standard. RMSE and correlation metrics are often used to evaluate the performance of the method, since it is possible to compare the resulting signal to the gold standard. With simulated signals, it is also possible to compare noise quantification metrics (e.g., signal-to-noise ratio (SNR), maximum drop in power density (DPR), power spectrum deformation ratio (Ω), etc.) for the noiseless signal, the signal with added noise and the denoised signal.

On the other hand, real signals are more representative of the reality. In some studies, the performance was evaluated with both simulated and real signals. The performance has generally been observed to be much lower with real signals [61]. As we have no gold standard, the noise quantification metrics of the measured signal and the denoised signal can also be compared. However, it is not possible to precisely quantify the noise that is still present in the denoised signal. Thus, it is more common to compare the performance of the proposed method to the state of the art. Alternatively, the performance of the methods using real signals can also be evaluated in terms of percentage of classification accuracy when the EMG is used in pattern recognition. The accuracy of the classification is then compared before and after denoising. Another method is also to study the remaining signal spectrum and compare it to before. Two issues can then be assessed simultaneously: 1. how much noise we managed to remove (e.g., 0–20 Hz) and 2. how much of the important information we managed to retain (20–500 Hz).

## 5. Discussion

The different methods of reducing the noise contamination found in the literature may be more or less suitable depending on the contaminant(s) present in the signal and on the requirements of the application. For example, there may be a need to process the EMG signal in real-time. In this case, the contaminant reduction method requires fast computation. In the same way, some applications of EMG may need to be pefrormed in controlled environments, while others must be sufficiently robust for use in real-life situations. Indeed, in controlled environments, especially for the acquisition performed over short intervals, the method does not necessarily need to take into account the variations in contaminant characteristics. In contrast, for real-life applications, it is more critical to use a method that is adaptable to the current contamination source. Finally, while the choice of method depends on the type of contaminant, it is also common to use more than one method to eliminate multiple contaminants simultaneously. A discussion regarding these main considerations is presented below.

### 5.1. Contaminant Type to Be Eliminated

Since contaminants all have different characteristics, it is not possible to identify a single method that works well for all contaminant types. For example, regression–subtraction is only suitable for PLI elimination since it requires the contaminant to be modelled as a sine wave. The same thing happens with template–subtraction, which has been developed specifically for ECG removal. Although there is no single method that can be optimized for all contaminants, we can identify the most effective and most used noise reduction approaches for certain types of contaminants. The adaptive noise canceller is an effective method for interference. However, it is not suitable for other contaminant types, such as artifacts and noise, because it requires the contaminant to be defined as a waveform in the time domain. Despite the number of studies examining the removal of ECG from the EMG signal, the current standard approaches are to either analyse only EMG signals between QRS complexes, where ECG contamination is considered negligible [12], or to use any of the template subtraction approaches [79]. However, other promising avenues using the WT and EMD have been proposed [79,93,97]. Ref. [79] compared different methods for ECG removal and concluded that there is no single approach that is optimal for all applications. Template–subtraction seems to achieve less distortion, which is required for fatigue analysis. However, the wavelet-based, EMD-based and high-pass methods provided better estimations of the signal’s amplitude, making them more suitable for applications that use force quantification. For WGN removal, Wavelet denoising is by far the most used approach. However, this method has been criticized due to the large number of parameters that must be set a priori. VMD and EMD methods have thus been proposed as alternatives. Ref. [106] compared their VMD-based methods to both EMD-based and wavelet-based methods in terms of SNR and RMSE and demonstrated that both proposed methods performed better. Moreover, they demonstrated that the VMD-SIT versions were more effective than VMD-WST, in addition to producing a smoother continuous signal. Ref. [108] also performed a comparative study of the VMD, EMD, EEMD and conventional IRR filtering methods for removing WGN. They showed that the VMD-based method performed better for removing WGN. Thus, the VMD-based method appears to be a promising avenue that merits further study. As for the reduction of the power-line interference, although the notch filter is not recommended for use in most applications [6], it appears to be a commonly used method in the literature. However, the adaptive noise canceller seems to be a great alternative despite its high computation complexity. The spectral interpolation proposed by [65] also presents a simple and promising alternative that allows for minimal alteration of the EMG spectrum.

### 5.2. Possibility to Be Used in Real-Time

Although some methods are preferred for some noise types, they have not necessarily been adapted to the applications in which they have been used. In some cases, such as prosthetic control and injury prevention, computation must be performed in real-time (i.e., at the same time as acquisition). Therefore, methods that require having the whole signal beforehand are not suitable for this kind of application. This is the case for both regression–subtraction and estimating the PLI using the least squares algorithm on the whole signal. Moreover, there are methods in which the parameters are optimized by hand according to the data, which is not possible to do in real-time. Finally, the calculation time and memory required in the method also play a role in its suitability. ANC, for example, has been criticized for its excessively long computation times because it requires an adjustment of every weight for each new sample, making it difficult to implement in real-time. Artificial networks such as ANFIS have recently been used to remove noise from contaminated EMG signals. ANFIS can be trained only once before they are ready for online testing. However, the need for an additional sensor to provide reference input is one of the drawbacks of these methods. With respect to decomposition methods, Ref. [117] tested the computational complexity of the EMD/EEMD and demonstrated that the result was equivalent to the Fourier transform. In addition, ref. [106] advocates that although the execution times of the VMD-SIT and VMD-WST are higher than the EMD and Wavelet, these methods can still be used in real-time.

### 5.3. Adaptivity of the Methods

Another issue that needs to be considered when choosing a contamination reduction method is the need to adapt it to the environment. In some cases, a reference noise signal is recorded at the beginning of the trial. This method allows for the adaptation of some of its parameters according to the noise currently present. However, this approach assumes that the signal will remain the same throughout the trial. In controlled environments and for trials performed over a long period, this assumption can be close to the reality, but in real-life applications, approaches using this scheme are not recommended. Further, some methods presented in this review can be modified to be a little more adaptive. For example, in Wavelet thresholding, the noise estimate ($\hat{\sigma }$) can be reevaluted throughout the trial and the thresholds adjusted accordingly. Finally, the most adaptive method is ANC, which adjusts its weights at each time step.

### 5.4. Complementarity of the Methods

Although some methods proved to be more effective than others, in some cases, more than one method was used in a complementary way for denoising. In fact, since some methods seem more effective for one particular type of noise, they may be combined with other methods that are effective for other types of noise. For example, [58] used wavelet thresholding to remove the remaining noise from its ANC. It is the same for [19], which uses the WT to remove the WGN and then the EEMD for the PLI and BW. Moreover, it is common to apply a band-pass filter (10–500 Hz) before the proposed denoising method [106]. According to [100], band-pass filtering allows for the reduction in the signal’s bandwidth, thus increasing the frequency resolution when the signal needs to be decomposed.

## 6. Conclusions

In this review, we have presented only methods that allow for the total reconstruction of the EMG signal. This means that after noise reduction processes have been applied, the result is a noise-free signal in the time domain that can be used as much for control and pattern recognition as for detecting fatigue or detecting myopathies. These methods are “optimal,” meaning they ensure that as much of the information contained in the EMG signal as possibe is retained. Certain application-specific methods exist that have not been presented here. In some cases, these methods can be further adapted to the specific needs of the application. For example, in proportional control, it is common to denoise the envelope of the signal rather than the signal itself. Moreover, the use of high-dimension EMG (HD-EMG) is growing and new HD-EMG-specific contamination reduction methods are being developed which require the use of more than one channel at a time. Ultimately, the choice of method will depend on the specific needs of the application and the characteristics of the contaminants present in the signal.

## Data Availability

Not applicable.

## Abbreviations

The following abbreviations are used in this manuscript:

ANC	Adaptive noise canceller
ANFIS	Adaptive neuro fuzzy inference system
BL	Baseline noise
BN	Background noise
BPN	Back propagation network
BSS	Blind source separation
BW	Baseline wander
CCA	Canonical correlation analysis
CCN	Cascade correlation network
CEEMD	Complementary ensemble empirical mode decomposition
CWT	Continuous wavelet transform
DCT	Discrete cosine transform
DPR	Maximum drop in power density
DWT	Discrete wavelet transform
ECG	Electrocardiographic signal
EEMD	Ensemble empirical mode decomposition
EMD	Empirical mode decomposition
EMG	Electromyography
ESAIC	Event-synchronous adaptive interference canceller
FIR	Finite impulse response
FT	Fourier transform
HD-EMG	High-dimension Electromyography
ICA	Independent component analysis
IMFs	Intrinsic mode functions
IT	Interval thresholding
IWT	Inverse wavelet transform
LMS	Least mean squares
MA	Motion artifacts
PCA	Principal component analysis
PLI	Power-line interference
RLS	Recursive least square
SCICA	Single channel independent component analysis
sEMG	Surface Electromyography
SER	Signal-to-ECG ratio
SIT	Soft interval thresholding
SMR	Signal-to-motion artifact ratio
SNR	Signal-to-noise ratio
SPR	Signal-to-powerline ratio
SWT	Stationary wavelet transform
VMD	Variational mode decomposition
VMFs	Variational mode functions
WGN	White Gaussian noise
WPT	Wavelet packet transform
WT	Wavelet transform
